# COVID-19 vaccine acceptance among pregnant women: a multicentre cross-sectional survey in Port Harcourt, Nigeria

**DOI:** 10.11604/pamj.2024.47.72.37446

**Published:** 2024-02-20

**Authors:** Hannah Emmanuel Omunakwe, Mary Okuku, Simeon Chijioke Amadi, Alali Dan-Jumbo

**Affiliations:** 1 Department of Haematology and Blood Transfusion, Rivers State University/Rivers State University Teaching Hospital, Port Harcourt, Nigeria,; 2 Department of Nursing Sciences, Rivers State University, Port Harcourt, Nigeria,; 3 Department of Obstetrics and Gynaecology, Rivers State University/Rivers State University Teaching Hospital, Port Harcourt, Nigeria,; 4 Department of Family Medicine, Rivers State University/Rivers State University Teaching Hospital. Port Harcourt, Nigeria

**Keywords:** COVID-19, vaccine, hesitancy, pregnancy, women, Port Harcourt, Nigeria

## Abstract

**Introduction:**

effective COVID-19 vaccines for the prevention of severe illness have been available for more than one year now. This study was carried out to ascertain vaccine hesitancy and its associations among pregnant women receiving antenatal care in Port Harcourt, a large cosmopolitan town in Nigeria.

**Methods:**

we conducted a cross-sectional online survey over 2 months among consenting pregnant women receiving antenatal care in the 3 largest obstetric service centers in Port Harcourt to evaluate COVID-19 vaccine hesitancy and its associations.

**Results:**

the prevalence of vaccine hesitancy was 669 (72.2%). Of the respondents, 27 (2.9%) had been infected or had a close family member infected with SARS-CoV-2, and 897 (96.8%) of them had heard of the COVID-19 vaccine; however, only 133 (14.4%) had been vaccinated against COVID-19. The safety of the mother in 260 (32.8%) and the safety of the unborn baby in 114 (14.4%) of the respondents were the reasons for vaccine hesitancy. A small proportion of women 7(0.9%) were hesitant on religious grounds. Tertiary education, use of childhood immunization for previous infants delivered, and availability of COVID-19 vaccine in the antenatal clinic at no cost to the women, were statistically significant predictors of vaccine uptake among the respondents.

**Conclusion:**

the prevalence of vaccine hesitancy among pregnant women in Port Harcourt was 72.2%. Higher academic achievement and availability of the COVID-19 vaccine in the antenatal clinic were predictors of vaccine uptake, while reasons for hesitancy were mostly due to safety concerns for the mother and unborn baby.

## Introduction

COVID-19 was declared a pandemic in March 2021 by the World Health Organization. Infection prevention modalities such as the use of facemasks, hand hygiene, and respiratory hygiene have been effective in preventing the spread of the infection, however in the last 1 year effective vaccines have been developed to prevent the infection and reduce the severity of the infection in people that are exposed. About 62% of the world´s population has been vaccinated, the majority of those vaccinated are in developed countries, and only 12% of the Nigerian population has been vaccinated at the time of this report.

According to WHO (2022), equitable access to effective and safe vaccines is critical to ending the COVID-19 pandemic. However, in addition to people being vaccinated, there is still a need to ensure good ventilation indoors, avoidance of crowds and maintaining physical distancing, wearing of masks, and performance of hand hygiene to control the spread of COVID-19 disease. Pregnancy was not found to predispose to a higher risk for COVID-19 infection as compared to non-pregnant women, however symptomatic pregnant women had more adverse outcomes than non-pregnant women [1]. COVID-19 infection is associated with high rates of preterm and cesarean births [2] as well as increased risk of fetal mortality [3]. Some studies in Nigeria have evaluated the willingness of Nigerians to receive the COVID-19 vaccine to be between 50-60% [4,5]. Many factors are linked with COVID-19 vaccine hesitancy in our society, but the myths surrounding its use in pregnancy have to do with claims that the vaccine causes infertility. This study sought to ascertain the prevalence of vaccination among pregnant women and the reasons for COVID-19 vaccine hesitancy among pregnant women in Port Harcourt.

## Methods

**Study design and study setting:** this was a cross-sectional prospective study conducted in the three large obstetric centers in Port Harcourt, Nigeria (Rivers State University Teaching Hospital, University of Port Harcourt Teaching Hospital, Obio-Cottage Health Centre) between January 14^th^ 2022 to March 18^th^ 2022, among pregnant women who were receiving antenatal care. The Port Harcourt is a metropolitan city in the Rivers State of Nigeria. The Rivers State is a core Niger Delta state in Nigeria. The health facilities in Port Harcourt, especially the tertiary ones, receive referrals from neighboring states. Rivers State has over five million residents and it is the sixth most populous state in Nigeria with a land mass of about 11,077 km^2^ (4,277 square meters).

**Inclusion/exclusion criteria:** pregnant women attending antenatal care in the study centers were included in the study. Those who did not give consent were excluded from the study.

**Data collection:** six research assistants were recruited and trained for the study. The research assistants recruited the patients who met the inclusion criteria daily from the antenatal clinic. The recruited participants were counseled and informed consent was obtained. The data for this study was collected using an adapted, structured, electronic questionnaire [6-8] administered once to each participant who could fill out the form on their device. For those who consented to participate but did not have a smartphone, a trained research assistant administered the questionnaire from his tablet and assisted the participant in filling and submit. The questionnaire contained seventeen questions about the socio-demographics characteristics, educational status, obstetric history, vaccination history, knowledge of COVID-19, and willingness to receive the COVID-19 vaccination.

**Sample size determination and sampling techniques:** the sample size was calculated using the formula:


$$n=\frac{z^{2}p\left(1-p\right)}{d^{2}}$$

Where, n = minimum sample size, Z = the standard normal deviation of 95% confidence level. This corresponds to 1.96. P = prevalence of COVID-19 vaccine acceptance in the antenatal women in Nigeria from previous studies = 33.8% (0.338) [7], d = level of precision (0.05). The minimum sample size was thus calculated to be 343. Given allowance for a 10% attrition rate (non-response rate), the adjusted sample size for the study was therefore 378 women. A total of 927 consenting pregnant women were however recruited through the simple random sampling method by balloting after the selection of the health facilities using the purposive sampling method.

**Statistical methods:** data was collated and analyzed on a Statistical Package for Social Sciences software (SPSS) version 23. Simple proportions were used in the descriptive analysis. Quantitative data were summarized and presented as mean and standard deviation while qualitative data were presented as numbers and percentages. The Chi-square test was used to compare categorical variables with a p-value of 0.05 or less taken as being statistically significant.

**Ethical approval:** it was obtained from the Hospital Ethics Committee of the Rivers State University Teaching Hospital. The ethical approval number was RSUTH/REC/2022/175.

## Results

**Socio-demographic characteristics:** in total, we had nine hundred and twenty-seven respondents within the study period. The majority of the respondents 297 (32.3%) were 31-35 years. The majority of the respondents 556 (60%) had completed tertiary education, 891 (96.2%) were married, and 494 (53.3%) had partners with a tertiary level of education. Most of them were self-employed 587 (63.3%) and had 1-3 people in their households as shown in Table 1.

**Table 1:** socio-demographic characteristics

Variable	Categories	Frequency; n = 927	Percent
**Age group (in years)**	< 20	10	1.1
	21 - 25	105	11.3
	26 - 30	276	29.8
	31 - 35	297	32.0
	36 - 40	191	20.6
	>40	48	2.2
**Marital status**	Single	18	1.9
	Married	891	96.2
	Chose not to say	18	1.9
**Highest education of the respondent**	No formal education	28	3.0
	Primary	7	0.8
	Secondary	336	36.2
	Tertiary	504	54.4
	Postgraduate	52	5.6
**Highest education of partner**	No formal education	57	6.1
	Primary	5	0.5
	Secondary	293	31.6
	Tertiary	494	53.3
	Post-graduate	78	8.4
**Occupation of respondent**	Civil servant	141	15.2
	Public Servant	118	12.7
	Business	462	49.8
	Self-employed	125	13.5
	Unemployed	81	8.7
**Occupation of partner**	Civil servant	223	24.1
	Public servant	200	21.6
	Business	393	42.4
	Self-employed	94	10.1
	Unemployed	17	1.8
**Number of persons in a household**	1 - 3	527	56.9
	4 - 6	358	38.6
	7 - 9	36	3.9
	≥ 10	6	0.6

**Figure 1:**
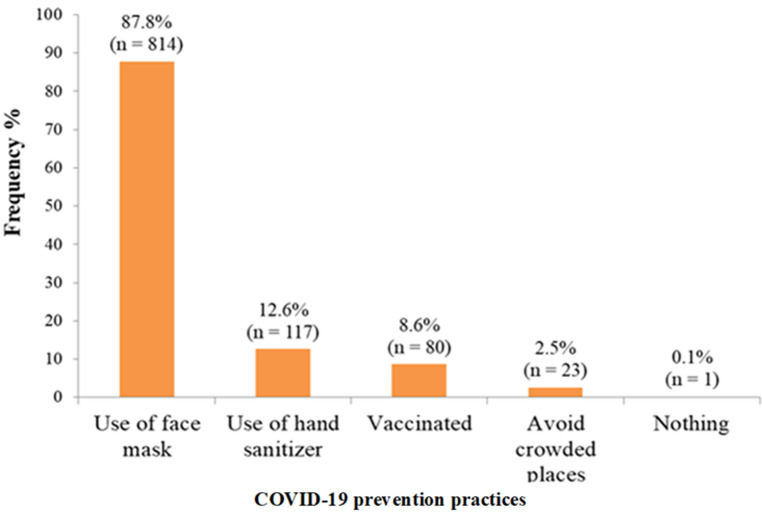
COVID-19 prevention practices carried out by respondents

**COVID-19 prevention practices carried out by respondents:** majority of the respondents 814 (87.8%) used Face masks and 117 (12.6%) used hand sanitizers for the prevention of COVID-19. One person (0.1%) admitted that she did nothing to prevent COVID-19 as shown in Figure 1.

**Pregnancy, antenatal care, and immunization history of respondents:** twenty-seven (2.9%) of the respondents had or a close family member tested positive for COVID-19 during the pandemic, and the majority 897 (96.8%) had heard of COVID-19 vaccination. However, only 133 (14.4%) had been vaccinated against the virus. Most of the respondents had been vaccinated for Tetanus in the index pregnancy 785 (84.7%). The majority of the parous women had vaccinated their infant for childhood vaccine-preventable diseases, however, 14 (2.3%) had not vaccinated their infants in the past. Of the 927 respondents, 222 (23.9%) said they would be willing to receive the COVID-19 vaccine if it was free and commonly available in the antenatal clinic as shown in Table 2.

**Table 2:** pregnancy, antenatal care (ANC), and immunization history of the respondent

Variable	Categories	Frequency; n = 927	Percent
Number of pregnancies carried to delivery	Nil	304	32.8
	One	277	29.9
	Two	223	24.1
	Three and above	123	13.3
			
Have had tetanus toxoid in recent pregnancy	Yes	785	84.7
	No	142	15.3
			
Took other children for routine immunization	Yes	681	73.5
	No	246	26.5
			
Will take COVID-19 vaccine if free and commonly available in ANC Clinic	Yes	222	23.9
	No	669	72.2
	Maybe	36	3.9

**Reasons for not taking COVID-19 vaccine:** the main reason for COVID-19 vaccine hesitancy among the respondents was the concern for the safety of the unborn baby, and so many wanted to take the vaccine after delivery as shown Table 3.

**Table 3:** reasons for not taking COVID-19 vaccine

Variable	Categories	Frequency	Percent
**Reasons for not taking COVID-19 vaccine (multiple response, n = 814)**	It’s not safe for me	260	32.8
	It’s not safe for the unborn baby	185	23.4
	Will take it when the vaccine is available	114	14.4
	Will take after delivery	88	11.1
	I will not be infected	26	3.3
	Fear of side effects of COVID-19 vaccine	36	4.5
	Fear due to conspiracy theory about COVID-19	58	7.3
	It’s against my religious belief	7	0.9
	Doubts/indecisive	13	1.6
	I will never take it	23	2.9
	COVID-19 doesn’t infect pregnant women	4	0.5

**Predictors of COVID-19 vaccine uptake:** on bivariate analysis, the predictors of vaccine uptake in the respondents were younger age <35 years, marital status, formal education, and having employment. However, logistic regression showed that achievement of tertiary education, use of childhood immunization for previous infants delivered, and availability of COVID-19 vaccine in the antenatal clinic at no cost were significant predictors of vaccine uptake in the respondents as shown in Table 4 and Table 4.1.

**Table 4:** predictors of COVID-19 vaccine uptake

Variable	COVID-19 vaccine uptake		Bivariate analysis	Logistics regression
	Yes	No	X^2^ (p-value)	OR (95%CI)
**Age group (in years) merged**				
< 20 - 25	23 (17.3)	92 (11.6)		0.59 (0.226 -1.557) p=0.289
26 - 35	61 (45.9)	512 (64.5)	16.745 (<0.001)	1.27 (0.679 - 2.387) p=0.451
36 - ≥40 (ref)	49 (36.8)	190 (23.9)		0
**Marital status**				
Single	6 (4.5)	12 (1.5)		1.10 (0.089 - 13.537) p=0.941
Married	116 (87.2)	775 (97.6)	38.591 (<0.001)	2.13 (0.223 - 20.283) p= 0.511
Chose not to say (ref)	11 (8.3)	7 (0.9)		0
**Highest education of the respondent**				
No formal education/primary	21 (15.8)	14 (1.8)		0.98 (0.262 - 3.647) p= 0.972
Secondary	13 (9.8)	323 (40.7)	94.790 (<0.001)	3.29 (1.445 - 7.472) p=0.005
Tertiary/post-graduate (ref)	99 (74.4)	457 (57.6)		0
**Highest education of partner**				
No formal education/primary	49 (36.8)	13 (1.6)		0.03 (0.009 - 0.117) p <0.001
Secondary	13 (9.8)	280 (35.3)	236.259 (<0.001)	1.09 (0.474 - 2.493) p= 0.820
Tertiary/post-graduate (ref)	71 (53.4)	501 (63.1)		0
**Occupation of respondent**				
Civil servant/public servant	61 (45.9)	198 (24.9)		0.64 (0.236 - 1.728) p=0.377
Business/self-employed	62 (46.6)	525 (66.1)	24.965 (<0.001)	1.12 (0.424 - 2.959) p=0.820
Unemployed (ref)	10 (7.5)	71 (8.9)		0
**Occupation of partner**				
Civil servant/public servant	78 (58.6)	345 (43.5)		9.11 (2.144 - 38.746) p=0.003
Business/self-employed	48 (36.1)	439 (55.3)	23.715 (<0.001)	17.26 (3.823 - 77.929)
Unemployed (ref)	7 (5.3)	10 (1.3)		0
**Number of persons in a household**				
1 - 3	75 (56.4)	452 (56.9)		
4 - 6	56 (42.1)	302 (38.0)	0.354*	
7 - 9	2 (1.5)	34 (4.3)		
≥ 10	0	6 (0.8)		

* = Fishers exact

**Table 4.1:** predictors of COVID-19 vaccine uptake

Variable	COVID-19 vaccine uptake		Bivariate analysis	Logistic regression
	Yes	No	X^2^ (p-value)	OR (95%CI)
**Have had tetanus toxoid in recent pregnancy**				
Yes	119 (89.5)	666 (83.9)	2.749 (0.097)	
No	14 (10.5)	128 (16.1)		
**Took other children for routine immunization**				
Yes	112 (84.2)	569 (71.7)	9.201 (0.002)	0.76 (0.396 - 1.438) p= 0.392
No	21 (15.8)	225 (28.3)		0
**Will take COVID-19 vaccine if free and commonly available in antenatal care (ANC) clinic**				
Yes	109 (82.0)	113 (14.2)		
No	23 (17.3)	646 (81.4)	<0.001*	
Maybe	1 (0.8)	35 (4.4)		

* = Fishers exact

## Discussion

The COVID-19 Pandemic ravaged the world from 2019 to date and despite the approval of the COVID-19 vaccine for use even in pregnancy, there has been a global hesitancy of acceptance of the vaccine by the parturients [9,10]. In this study, the COVID-19 vaccine acceptance rate by pregnant women who had not been vaccinated was 23.9%. This is lower than findings from other studies in Ethiopia (70.9%) [11], Addis Ababa (80.9%) [12], China (91.3%) [13], Indonesia, Southeast Asia (93.3%) [14], South Africa (81.6%) [15], Saudi Arabia (64.7%) [16], United States (67%) [17], but similar to findings from the studies in Middle Eastern Populations (36.8%) [18], Poland (31.3%) [19], Turkey (37%) [20] and Kano in Nigeria (33.8%) [6]. The observed difference may be due to the differences in access to healthcare services in the different study areas, awareness of the severity of COVID-19, the number of fatalities due to COVID-19 in the different study populations, and other study population differences.

Tertiary level of education was found to be significantly associated with uptake of the COVID-19 vaccine among respondents. This is similar to the finding in a survey of 664 women in France, where higher education achievement was associated with willingness to receive the vaccine [21]. This is at variance with the findings from studies in Ethiopia and South Africa where primary level of education was found to be associated with acceptance of the COVID-19 vaccine [3,22]. The difference may be due to the variations in the availability of news and access to social media.

The reasons for the non-acceptance of the COVID-19 vaccine found in this study were safety concerns for the parturient and her unborn baby and the unavailability of the vaccine. This is like findings from other studies in Ethiopia and Northern Nigeria [3,13]. The awareness level of COVID-19 and its preventive measures among respondents in this study was high Figure 1. This is similar to the findings in the study in Ethiopia [3]. This similarity may be because the two studies were carried out among pregnant women who routinely undergo antenatal classes and have regular health enlightenment from their attending physicians and nurses on COVID-19 and other health-related issues. This finding is however, higher than findings from other studies conducted among Gondar City residents, in Northern Ethiopia (50.7%) [23], and in Dirashe district, Southern Ethiopia (63.5%) [24]. The variation may be because participants in these studies did not have as many health education opportunities as the pregnant women in our study as those studies were carried out in the general population.

The major preventive measures against COVID-19 adopted by the respondents in this study were the use of face masks and use of hand sanitizers. The adoption of preventive measures by a large percentage of the respondents in this study is higher than the results from the studies in Gondar City in Ethiopia [15] and in South Africa [25]. The differences found in these studies may be because pregnant women routinely visit the hospitals for antenatal where they are taught and mandated to adopt COVID-19 preventive measures for their safety and that of the health workers. Respondents in the other studies may not have as many opportunities for COVID-19 education and application of such knowledge as pregnant women.

**Limitation:** this study was carried out in an urban area in Southern Nigeria. This notwithstanding, Port Harcourt is a metropolitan city with people from different tribes and socio-economic statuses. And so, within the limits of acceptable error, the findings of this study should be reflective of the general population.

## Conclusion

In this study, the COVID-19 vaccine acceptance level among pregnant women in Port Harcourt, Nigeria was low. Tertiary level of education was found to be significantly associated with COVID-19 vaccine acceptance in pregnancy and there was high awareness of COVID-19 and its preventive measures among the parturient in this study. Safety concerns for both the mother and the baby were the main reason for the high level of COVID-19 vaccine hesitancy found in this study. Steps should be taken by the government and all stakeholders to make the COVID-19 vaccine available in routine antenatal services and educate the women and their families on the benefits, efficacy, and safety of the vaccine to improve the acceptability of the vaccine among the pregnant population in our setting.

### What is known about this topic

- *The COVID-19 vaccine acceptance rate in the Nigerian general population was low;*
- *The COVID-19 actual vaccination rate in the Nigerian general population was low with limited data on the pregnant population*.

### What this study adds

- *This study has provided knowledge on the COVID-19 vaccine hesitancy among pregnant women in Port Harcourt, Nigeria, and the plausible reasons why they do not accept the vaccine*.
